# Supplementary material for: Causal interplay between lactose intolerance and gut microbiota: a combined bidirectional Mendelian randomization and in vivo validation study
Source: Front Nutr. 2026 Jun 1;13:1803337. doi: 10.3389/fnut.2026.1803337 (PMC13265576; doi:10.3389/fnut.2026.1803337)
Supplement: Supplementary file 1 [file Data_Sheet_1.zip › supplementary materials/Forward/leave one out cross validation/ebi-a-GCST90027805.finngen_R12_E4_LACTONAS.pdf]

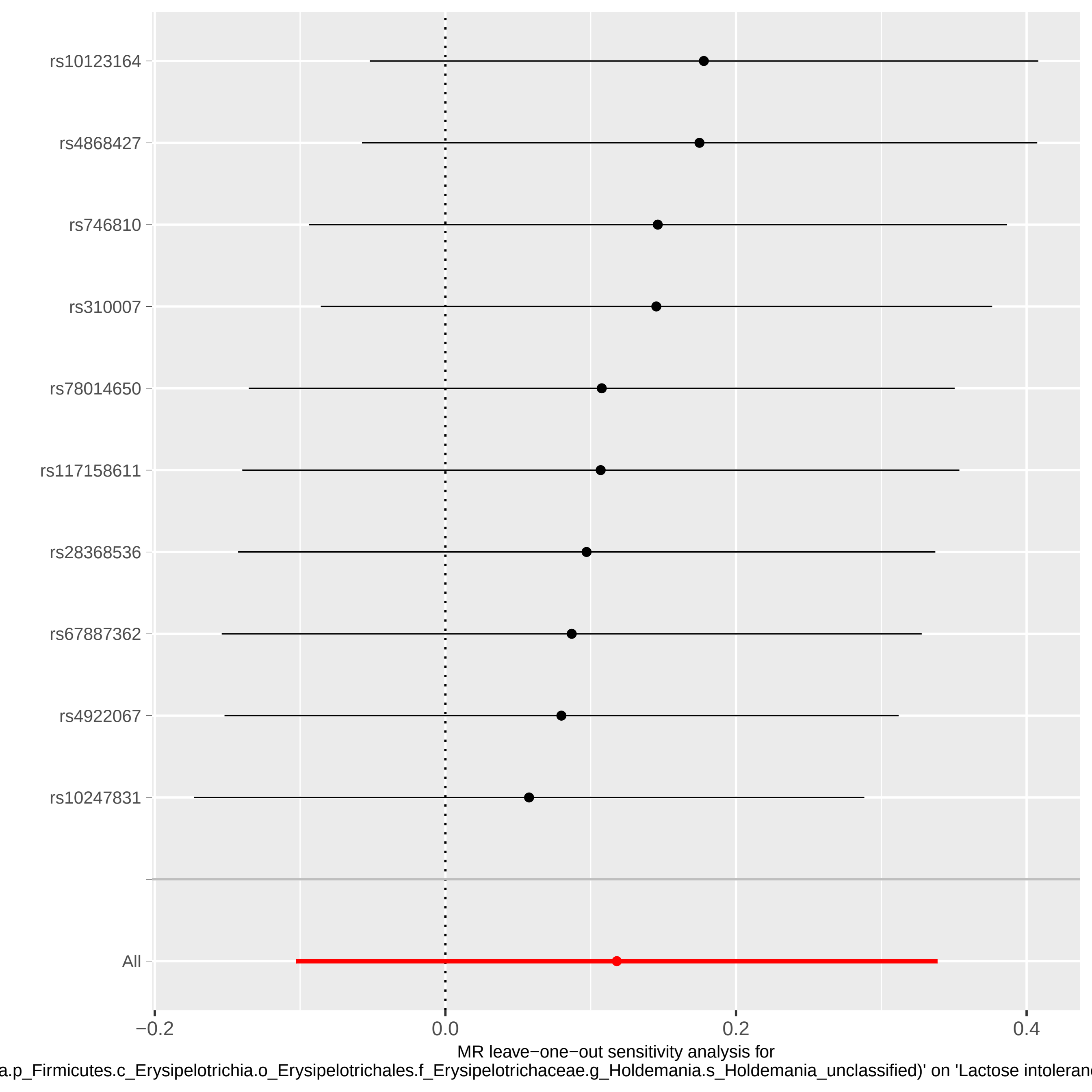

rs10123164
rs4868427
rs746810
rs310007
rs78014650
rs117158611
rs28368536
rs67887362
rs4922067
rs10247831
All
−0.2
0.0
0.2
0.4
MR leave−one−out sensitivity analysis for
a.p_Firmicutes.c_Erysipelotrichia.o_Erysipelotrichales.f_Erysipelotrichaceae.g_Holdemania.s_Holdemania_unclassified)' on 'Lactose intoleran
